# Protective Roles of Sodium Butyrate in Lipopolysaccharide-Induced Bovine Ruminal Epithelial Cells by Activating G Protein-Coupled Receptors 41

**DOI:** 10.3389/fnut.2022.842634

**Published:** 2022-05-06

**Authors:** Tianyu Yang, Osmond Datsomor, Maocheng Jiang, Xiaoyu Ma, Guoqi Zhao, Kang Zhan

**Affiliations:** College of Animal Science and Technology, Institute of Animal Culture Collection and Application, Yangzhou University, Yangzhou, China

**Keywords:** sodium butyrate, bovine ruminal epithelial cells, G protein-coupled receptors 41, inflammation, volatile fatty acid metabolism

## Abstract

This study aimed to evaluate whether sodium butyrate (SB) attenuates the ruminal response to LPS-stimulated inflammation by activating GPR41 in bovine rumen epithelial cells (BRECs). We examined the SB regulation of GPR41 and its impact on LPS-induced inflammation using GPR41 knockdown BRECs. The LPS-induced BRECs showed increases in the expression of genes related to pro-inflammation and decreases in the expression of genes related to tight junction proteins; these were attenuated by pretreatment with SB. Compared with that in LPS-stimulated BRECs, the ratio of phosphorylated NF-κB (p65 subunit) to NF-κB (p65 subunit) and the ratio of phosphorylated IκBα to IκBα were suppressed with SB pretreatment. The LSB group abated LPS-induced apoptosis and decreased the expression of Bax, Caspase 3, and Caspase 9 mRNA relative to the LPS group. In addition, the LSB group had a lower proportion of cells in the G0–G1 phase and a higher proportion of cells in the S phase than the LPS group. The mRNA expression of ACAT1 and BDH1 genes related to volatile fatty acid (VFA) metabolism were upregulated in the LSB group compared to those in LPS-induced BRECs. In addition, pretreatment with SB promoted the gene expression of GPR41 in the LPS-induced BRECs. Interestingly, SB pretreatment protected BRECs but not GPR41KD BRECs. Our results suggest that SB pretreatment protects against the changes in BRECs LPS-induced inflammatory response by activating GPR41.

## Introduction

To meet the demand for dairy products, a high-concentrate diet has been increasingly fed to dairy cows to improve milk yield. However, a high-concentrate diet is detrimental to dairy cows when administered for long periods, which can reduce ruminal pH and microbial population and increase the risk of subacute ruminal acidosis (SARA; 1). The rumen is the first organ affected by low pH, and pH-related damages affecting the integrity and permeability of rumen epithelium have been reported (2–4). Penetration of the ruminal epithelial barrier by endotoxins and antigens is limited in the normal physiologic state (5). Unfortunately, a decrease in pH may prompt the translocation of lipopolysaccharide (LPS), affecting dairy-cow health and productivity (6, 7). Similar to exogenous infectious pathogens, LPS-release can elicit an inflammatory response in rumen epithelium (8). It has been reported that inflammatory molecules such as LPS elicit a severe inflammatory response in multiple types of cells, such as bovine rumen epithelial cells (BRECs), and activate the inflammatory NF-κB pathway (9). Activation of the NF-κB pathway can increase the expression of genes related to inflammatory response, including interleukin (IL)-1β, IL-6, and tumor necrosis factor (TNF)-α (10). In addition, NF-κB transcription factors are key regulators of apoptosis and differentiation. It has been reported that LPS can induce apoptosis and potently block proliferation (11, 12). Therefore, there is an urgent need to develop alternative therapeutic strategies to alleviate LPS-induced BREC injury.

The rumen contains a large amount of the ruminal microbiota, which can metabolize dietary carbohydrates into short-chain fatty acids (SCFAs), including acetate, propionate, and butyrate (13). Since butyrate has been demonstrated to show positive effects such as controlling enteric pathogens and reducing inflammation, particular attention has been paid to sodium butyrate (SB; 14). It has been reported that SB heightens anti-inflammatory, antioxidant, and antiapoptotic capacity in ruminant cell lines (14, 15). SB not only demonstrates a suppression of pro-inflammatory TNF-α, IL-6, and IL-1β (15), it can also facilitate alterations in the degradation or phosphorylation of IκBα and the dampening of NF-κB signaling (16). In addition, oral supplements with SB alleviate the negative effect of high-concentrate-diet-induced LPS-release on the rumen epithelium of dairy goats and decrease the secretion of cytokines (8). The same is true of liver inflammation, which is also alleviated by SB supplementation. In addition, infusions of SB are beneficial to modeling the rumen epithelial morphology by inhibiting apoptosis and affecting cell cycle progression. However, little information of the mechanism has been revealed, and SB has not previously been studied in LPS-stimulated BRECs.

Metabolic disorders appear to have a similar background to low-grade chronic inflammation, although the pathophysiological mechanisms leading to tissue and organ damage are not fully elucidated. Infusion of SB has been proposed to enhance SCFA uptake and metabolism in ruminal epithelial of goats (17). Moreover, SCFA uptake and BREC metabolism were altered by exposure to LPS (18). Previous studies have demonstrated that G-protein-coupled receptors 43 (GPR43) and 41 (GPR41) are receptors for SCFAs in humans (19–21). GPR41 and GPR43 are differentially expressed in various cell types, such as fat, neutrophils, and colonic epithelial cells, and they regulate host energy balance and immune responses (22–24). In addition, while the expression of GPR41 was detected in BRECs, GPR43 was not observed (13). Previous studies showed that SB is more active toward GPR41 than other SCFAs (25). We hypothesized that SB may regulate the inflammatory response via GPR41 in LPS-induced BRECs. Therefore, the objective of this study was to provide evidence for the protective roles of SB in LPS-induced BRECs by activating GPR41.

## Materials and Methods

All trials plan and methods have authorized with the Animal Ethics Committee of Yangzhou University, China. The study conducted under the Care and Use of Laboratory Animals guidelines.

### Cell Culture

The Institute of Animal Culture Collection and Application (IACCA), Yangzhou University was the supplier of both GPR41 knockdown BRECs (GPR41KD BRECs) and immortal BRECs (13). Immortal BRECs obtained from IACCA and utilized in this research were cultured in DMEM/F12 medium comprising of 10% FBS, 100 U/mL penicillin, 100 μg/mL streptomycin.

### Experimental Design

The LPS used in this study was from *Escherichia coli* O55:B5 lyophilized powder (L6529, Sigma-Aldrich, St. Louis, MO, United States).

**FIGURE 1:**
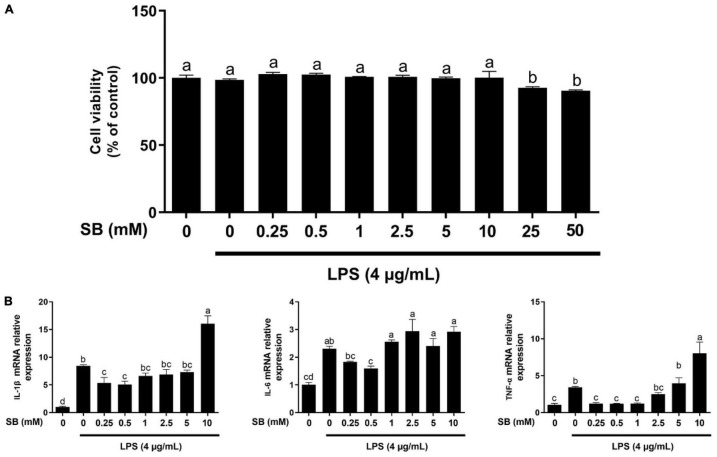
Effects of sodium butyrate (SB) on cell viability and gene expression of inflammatory cytokines. **(A)** Bovine rumen epithelial cells (BRECs) were treated with DMEM/F12 (Control group); or with different concentrations of SB (0, 0.25, 0.5, 1, 2.5, 5, 10, 25, and 50 mM) for 18 h, followed by a challenge with 4 μg/mL lipopolysaccharide (LPS) for an additional 6 h. Cell viability was determined using the Cell Counting Kit-8 Assay (Dojindo, Kumamoto, Japan). Values are shown as mean ± SEM (*n* = 6). **(B)** Bovine rumen epithelial cells were pretreated with different concentrations of sodium butyrate (0.25, 0.5, 1, 2.5, 5, and 10 mM) for 18 h and then treated with 4 μg/mL LPS for 6 h. The gene expression of IL-1β, IL-6, and TNF-α was analyzed by qRT-PCR, which are normalized to GAPDH. Values are shown as mean ± SEM (*n* = 3). Different lowercase letters in the bar chart indicate significant differences (*P* < 0.05).

Cell inflammatory models were established and optimized with respect to LPS and SB concentrations as well as the timing of inflammatory responses. BRECs were treated with either SB for 18 h for the SB group (SB) or with LPS for 6 h for the LPS group (LPS). BRECs were treated with medium without SB and LPS for the control group (CON). Experiments were performed using pretreatment with SB for 18 h followed by washing and then LPS exposure for 6 h for the LSB group (LSB), and GPR41KD BRECs pretreatment with SB for 18 h followed by washing and then LPS exposure for 6 h for the LSB-GPR41KD group (LSB-GPR41KD). In addition, GPR41KD BRECs were treated with medium without SB and LPS for the GPR41KD group (GPR41KD). Cells were exposed to optimized doses of 4 μg/mL LPS and 0.5 mM SB according to the results of a dose-dependence assay (Figure 1) in this study and results reported in other works (15).

### Cell Viability Assay

The cytotoxic effects of SB on LPS-induced BRECs were determined using the Cell Counting Kit-8 (CCK-8; Dojindo, Shanghai, China) according to the previous study (27). The BRECs were seeded into 96-well plates (5 × 10^3^ cells/well). After 12 h, the cells were cultured in DMEM/F12 medium in the presence or absence (control group) of varying concentrations of SB (0, 0.25, 0.5, 1, 2.5, 5, 10, 25, and 50 mM) for 18 h, followed by a challenge with 4 μg/mL LPS for 6 h (*n* = 6). After incubation, the cells were washed five times with 200 μL of sterile water and incubated with 100 μL of DMEM/F12 supplemented with 10 μL of CCK-8 at 37°C and 5% CO_2_ for 3 h. Absorbance at 450 nm was then measured in each well using an auto-microplate reader. Cell viability (%) = (treatment OD – blank OD)/(control OD – blank OD).

### Quantitative RT-PCR

For mRNA expression analysis, the BRECs were plated in 6-well plates at a density of 2 × 10^5^ cells/well. After incubation, total RNA was isolated from the cultured cells using FastPure Cell/Tissue Total RNA Isolation Kit (RC101, Vazyme Biotech Co., Ltd., Nanjing, China) in accordance with the manufacturer’s guidances. An OD-1000 + Micro-Spectrophotometer was used to measure RNA purity and concentration, and RNA quality was measured via electrophoresis (2% agarose gels). In our study, the optical density (OD) 260/OD280 ratio of the total RNA was determined to be 1.9, and the intensity of the 28S ribosomal RNA band was approximately twice that of the 18S ribosomal RNA band in total RNA samples, indicating that the total RNA was of high quality. Reverse transcription (RT) was performed using an RT kit (Takara, Beijing, China). The RT reaction mixtures contained 1 μg of total RNA and 1 × PrimeScript RT Master Mix in a final volume of 20 μL, and the reactions were performed for 15 min at 37°C. Reverse transcriptase was inactivated by heating to 85°C for 5 s. qRT-PCR assays were performed using the SYBR^®^ Premix Ex Taq™ II Kit (Takara). The qRT-PCR reaction mixture contained 1 × SYBR^®^ Premix Ex Taq™ II, of forward and reverse primers at 0.4 μM, and 100 ng of cDNA templates in a final volume of 20 μL, and the reactions were performed as follows: initial denaturation at 95°C for 30 s followed by 40 cycles at 95°C for 5 s and 60°C for 30 s. Before performing qRT-PCR on the samples, primers were designed to span exon–exon junctions, where possible, which were evaluated for dimer formation by generating melt curves following amplification to verify the presence of a single product. The primers used are listed in Table 1. The reaction of a negative control without a cDNA sample was performed. RefFinder,^1^ including Normfinder, geNorm, and the comparative ΔCT method, was used to select the first-rank reference gene (ACTB and GAPDH) by determining the ranking of the candidate genes. The final ranking was calculated by assigning an appropriate weight value to each gene, and the geometric mean of their weight values for the overall final ranking was confirmed. A higher expression stability was indicated by a lower gene geomean of ranking value. Eventually, GAPDH was screened for subsequent study. In addition, GAPDH is already known to be suitable for BRECs and GPR41KD BRECs (13). The quantitative PCR results were analyzed using the 2^–ΔΔ*Ct*^ method for calculating the fold changes in the mRNA levels of targeted genes (26). All trials were repeated three times.

**TABLE 1:** Primers for real-time PCR analyses.

Gene	Primer sequence, 5′–3′	Accession number	Size (bp)
GAPDH	F: GGGTCATCATCTCTGCACCT	NM_001034034	176
	R: GGTCATAAGTCCCTCCACGA		
IL-6	F: TCCTTGCTGCTTTCACACTC	NM_173923.2	129
	R: CACCCCAGGCAGACTACTTC		
IL-1β	F: CAGTGCCTACGCACATGTCT	NM_174093.1	209
	R: AGAGGAGGTGGAGAGCCTTC		
TNF-α	F: GCCCTCTGGTTCAGACACTC	NM_173966.3	192
	R: AGATGAGGTAAAGCCCGTCA		
ZO-1	F: TCTGCAGCAATAAAGCAGCATTTC	XM_010817146.1	187
	R: TTAGGGCACAGCATCGTATCACA		
Claudin-1	F: CGTGCCTTGATGGTGAT	NM_001001854.2	102
	R: CTGTGCCTCGTCGTCTT		
Occludin	F: GAACGAGAAGCGACTGTATC	NM_001082433.2	122
	R: CACTGCTGCTGTAATGAGG		
MCT1	F: CAATGCCACCAGCAGTTG	NM_001037319.1	376
	R: GCAAGCCCAAGACCTCCAAT		
MCT4	F: AGCGTCTGAGCCCAGGGAGG	NM_001109980.2	223
	R: ACCTCGCGGCTTGGCTTCAC		
ACAT1	F: CGGGTGCAGGAAATAAGGTA	NM_001046075.1	133
	R: TAGTGGCTGGCAGAG		
BDH1	F: GACCCTGAGAAAGGCTTGT	NM_001034600.2	92
	R: TCCTTGTAGGTCTCCATGC		
Caspase 3	F: GTGGATGCAGCAAACCTCAG	NM_001077840	288
	R: TCGGCAGGCCTGAATAATGA		
Caspase 9	F: TGTCTTGAATGCAGACCCCT	NM_001205504	190
	R: CAAAGCCTGGACCATTTGCT		
Bcl-2	F: ATGACCGAGTACCTGAACCG	NM_001166486	127
	R: CCTTCAGAGACAGCCAGGAG		
BAX	F: GGAGATGAATTGGACAGTAAC	NM_173894	120
	R: GTTGAAGTTGCCGTCAGA		
Cyclin D1	F: GCACTTCCTCTCCAAGATGC	NM_001046273	204
	R: GTCAGGCGGTGATAGGAGAG		
Cyclin D3	F: TCCAAGCTGCGCGAGACTAC	XM_005223490	178
	R: GAGAGAGCCGGTGCAGAATC		
CDK2	F: CTCACTGATCTTGTCTGGTT	NM_001014934	170
	R: TAAGCAACGACTAAGAGGAG		
CDK4	F: ACTCTGGTATCGTGCTCCAGAAG	NM_001037594	114
	R: CAGAAGAGAGGCTTTCGACGAA		
CDK6	F: TTCGTGGAAGTTCAGATGTC	NM_001192301	165
	R: TGCCTTGTTCATCAATGTCT		
ZO-1	F: TCTGCAGCAATAAAGCAGCATTTC	XM_010817146.1	187
	R: TTAGGGCACAGCATCGTATCACA		
GPR41	F: AACCTCACCCTCTCGGATC	NM_001145233.1	214
	R: GCCGAGTCTTGTACCAAAGC		

*F, forward; R, reverse.*

### Western Blotting Analysis

For protein expression analysis, BRECs were seeded in a 10 cm dish (2 × 10^6^ cells/well) and grown at 37°C and 5% CO_2_. After incubation, bovine hepatocytes were lysed to extract total protein in a RIPA lysis (Thermo Scientific, Shanghai, China) containing 1 × protease inhibitor cocktail (Thermo Scientific) and 1 × phosphatase inhibitor cocktail tablets (Roche, Shanghai, China). Protein concentrations were determined using a BCA kit (Beyotime, Beijing, China) according to manufacturer protocols. The target protein abundance in the BRECs was quantified following a published study (13). Equal amounts (40 μg) of protein lysates were fractionated by SDS-PAGE and transferred to nitrocellulose membranes (PALL, Shanghai, China). The membranes were blocked with 5% horse serum and then incubated with gentle shaking overnight at 4°C, with the primary antibody plus 5% horse serum in Tris-buffered saline with Tween (TBS-T: 10 mM Tris–HCl, pH 7.5, 150 mM NaCl, 0.05% Tween 20). The sources of the commercial antibody used in the experiment were GAPDH (1:1000; CST, Shanghai, China), phosphorylated (p)-p65, p65 (1:750; CST, Shanghai, China), p-IκBα, and IκBα (1:750, Affinity, Biotech Co., Ltd., Liyang, China). The horseradish peroxidase (HRP)-conjugated secondary antibody was used, which included goat anti-rabbit IgG (1:5,000; CST). The target bands were detected using the Super Signal West Femto Maximum Sensitivity Substrate or Pierce ECL Plus Western Blotting Substrate (Thermo Scientific).

### Staining by Fluorescein Isothiocyanate-Phalloidin

The staining by FITC-phalloidin was processed as described by manufacturer’s protocol. In brief, the cells were fixed with paraformaldehyde for 20 min and treated with 0.5% Triton X-100 solution for 5 min. Then, the cells were covered with 100 nM FITC-phalloidin solution (Affinity, Biotech Co., Ltd., Liyang, China) and incubated at room temperature away from light for 30 min. The nuclei were stained with 100 nM DAPI solution (Affinity, Biotech Co., Ltd., Liyang, China) for 3 min. Subsequently, the cells were examined and photographed under a fluorescence microscope (DMi8, Germany) with 496- and 364-nm light excitation, respectively.

### Measuring Apoptotic Cell Numbers by Flow Cytometry

The apoptotic effect on the BRECs was measured using an Annexin V-FITC/PI Apoptosis Detection Kit (A211, Vazyme Biotech Co., Ltd., Nanjing, China). The cells were collected using a 2.5% trypsin solution and stained by fluorescein isothiocyanate (FITC)/propidium iodide (PI). Next, all samples were analyzed for apoptosis by flow cytometry (BD FACSAria SORP, United States) and fluorescence microscopy (DMi8, Germany). FlowJo version 10.7.1 (BD Biosciences) software was used to evaluate the apoptosis rate of cells.

### Measuring Cell Cycle by Flow Cytometry

The cell cycle was determined using a Cell Cycle Quantitation Assay Kit (CA1510, Solarbio, China). The cells were collected using a 2.5% trypsin solution and fixed by 70% ethyl alcohol at 37°C for 2 h and 47°C for 12 h. The cells were collected by centrifugation and incubated with RNase at 377°C for 30 min. The cells were then stained at 47°C for 30 min with 400 μL PI. Subsequently, the BREC cell cycle was determined by flow cytometry (BD FACSAria SORP, United States) with 488-nm light excitation. FlowJo version 10.7.1 (BD Biosciences) software was used to analyze the proportion of cells at different proliferative stages.

### Statistical Analysis

The effect of GPR41 was analyzed using the T-test, and other results were evaluated by one-way analysis of variance (ANOVA), followed by determining the least significant difference (LSD) for *post hoc* multiple comparison of treatment means using SPSS 19.0 software (SPSS Inc.; Chicago, IL, United States). *P* Value < 0.05 were considered significant. *P* Value < 0.1 were considered trending.

## Results

### Effect of Sodium Butyrate Supplementation on Viability and the Adaptive Response to Inflammation of Bovine Rumen Epithelial Cells Challenged With Lipopolysaccharide

The relationship between SB content and its protective effect on BRECs challenged with LPS was examined by the CCK-8 assay. As shown in Figure 1, LPS showed no effect (*P* > 0.05) on cell viability compared with the control. Moreover, related to the LPS group, viability was not affected (*P* > 0.05) by pretreatment with SB from 0.25 to 10 mM on BRECs challenged with LPS; however, pretreatment with 25 and 50 mM SB inhibited (*P* < 0.05) BREC viability.

The mRNA levels of genes encoding the cytokines were analyzed by qRT-PCR (Figure 1B). Compared with the control, LPS alone enhanced (*P* < 0.05) the mRNA abundance of IL-1β, IL-6, and TNF-α. Pretreatment with 0.5 mM SB markedly decreased (*P* < 0.05) the gene expression of IL-1β, IL-6, and TNF-α compared to LPS alone. These results reveal that the doses of 4 μg/mL LPS and 0.5 mM SB did not have an effect on BRECs viability, and the inflammation model met the purpose of the study.

### Lipopolysaccharide-Induced Activation of the Expression of Proteins Related to NF-κB Signaling Suppressed by Sodium Butyrate Pretreatment

**FIGURE 2:**
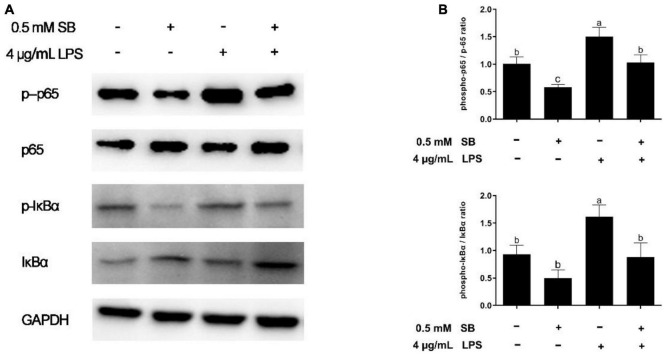
Sodium butyrate (SB) inhibiting the canonical NF-κB signaling pathway. BRECs were pretreat with SB (0.5 mM) for 18 h, followed by stimulation with LPS for 6 h. Western blotting was performed to determine the phosphorylation levels of IκB and p65. **(A,B)** Immunoblotting and acquisition of intensity from the respective blots. The values are shown as mean ± SEM (*n* = 3). The letters in superscript indicate that the difference between groups was significant (*P* < 0.05).

The phosphorylation levels of the NF-κB pathway related to proteins IκB and p65 were detected with Western blotting, as seen in Figure 2. Compared with the control group, LSP increased (*P* < 0.05) the ratio of phosphorylated NF-κB (p65 subunit) to NF-κB (p65 subunit) and the ratio of phosphorylated IκBα to IκBα (Figures 2A,B). For the LPS group, pretreatment with SB decreased (*P* < 0.05) the ratio of phosphorylated NF-κB (p65 subunit) to NF-κB (p65 subunit) and the ratio of phosphorylated IκBα to IκBα (Figures 2A,B), indicating that SB inhibited the canonical NF-κB signaling pathway.

### Effect of Sodium Butyrate Supplementation on the Expression of Genes Related to Tight Junction Proteins and Cytoskeleton of Bovine Rumen Epithelial Cells Challenged With Lipopolysaccharide

**FIGURE 3:**
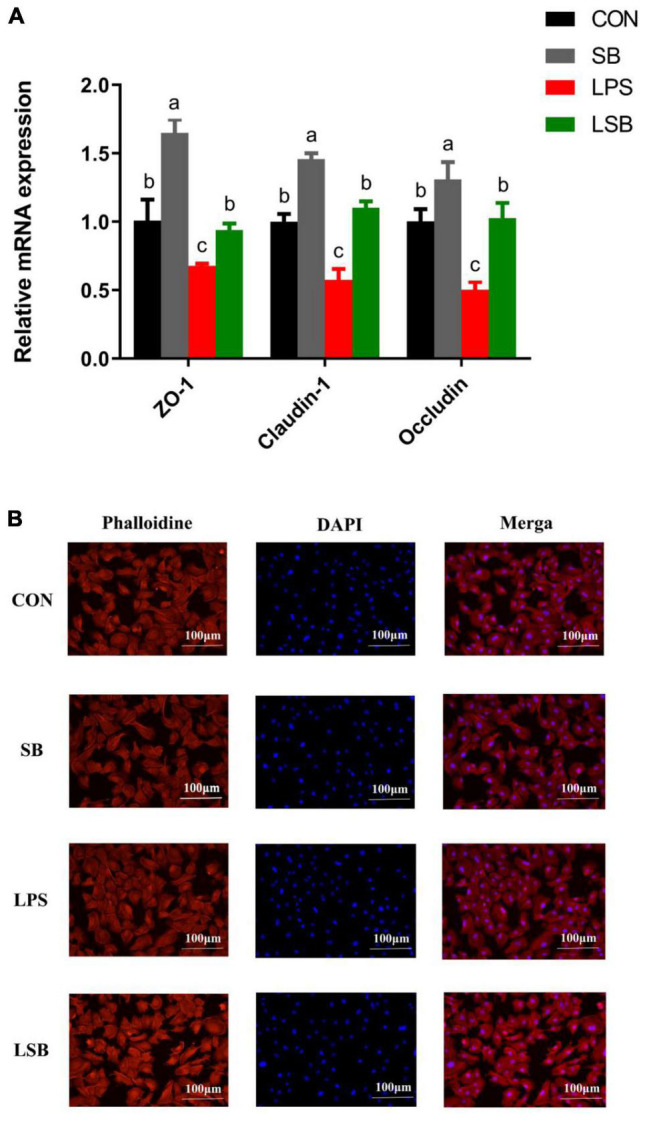
Effects of pretreatment with SB on the expression of genes related to tight junction proteins and cellular morphology in BRECs stimulated with LPS. BRECs were pretreated with SB (0.5 mM) for 18 h followed by stimulation with LPS for 6 h. **(A)** The gene expression of ZO-1, Claudin-1, and Occludin was analyzed by qRT-PCR. **(B)** Rhodamine phalloidine staining of BRECs. The values are shown as mean ± SEM (*n* = 3). The letters in superscript indicate that the difference between groups was significant (*P* < 0.05).

The expression of tight junction proteins is shown in Figure 3A. Compared to the control group, supplementation with SB increased (*P* < 0.05) the expression of ZO-1, Claudin-1, and Occludin, while LPS decreased (*P* < 0.05) the expression levels for the above-mentioned genes. However, these decreases were reversed (*P* < 0.05) in the LSB group. Furthermore, fluorescence results showed no effect on the cytoskeleton with the current treatment concentrations (Figure 3B).

### Sodium Butyrate Inhibits Apoptosis and Modulates Cell Cycle of Bovine Rumen Epithelial Cells Challenged With Lipopolysaccharide

**FIGURE 4:**
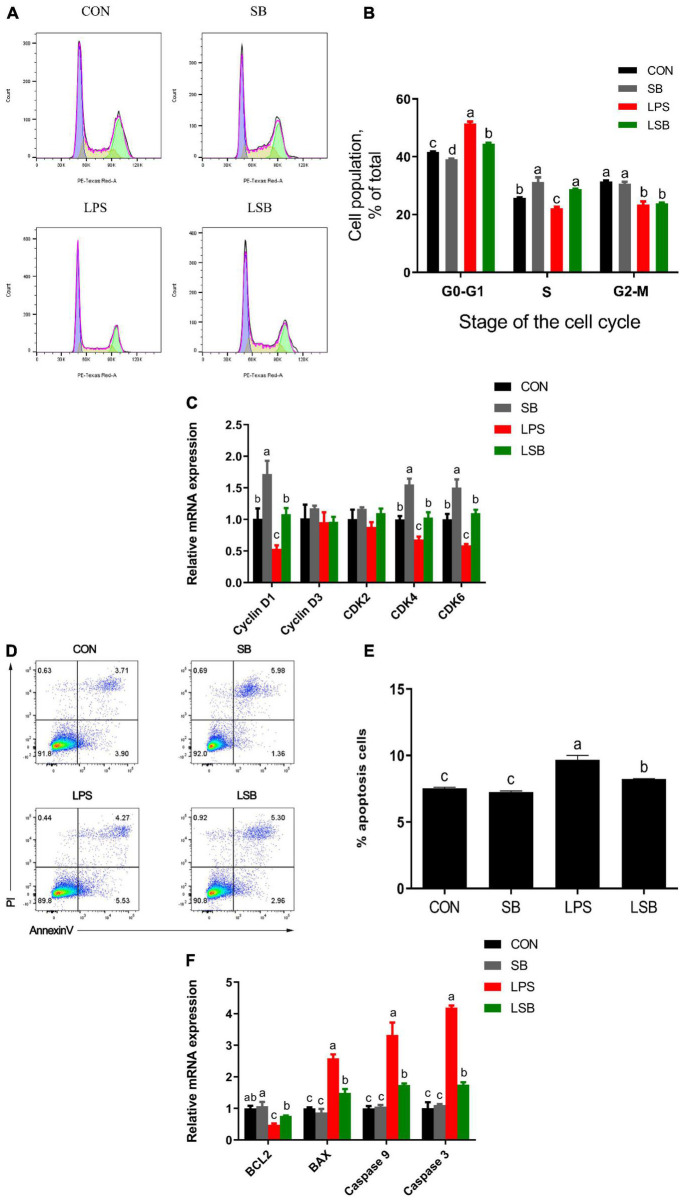
Effect of SB on proliferation and apoptosis in BRECs stimulated with LPS. BRECs were pretreat with SB (0.5 mM) for 18 h followed by stimulation with LPS for 6 h. After incubation, cells were analyzed by flow cytometry and qRT-PCR. **(A)** A representative histogram of flow cytometric analyses. **(B)** Percentages of cells at different phases of the cell cycle. **(C)** Gene expression of Cyclin D1, Cyclin D3, CDK 2, CDK 4, and CDK6. **(D)** Absorbance of FITC-A; absorbance of fluorescein isothiocyanate-Annexin V. **(E)** Apoptosis percentages in cultures exposed to LPS or SB. **(F)** Gene expression of Caspase 3, Caspase 9, Bcl-2, and BAX. The values are shown as mean ± SEM (*n* = 3). The letters in superscript indicate that the difference between groups was significant (*P* < 0.05).

To verify the effect of SB on the apoptosis and proliferation of BRECs challenged with LPS, we used flow cytometry and qRT-PCR to obtain reliable conclusions. Pretreatment with SB had fewer (*P* < 0.05) cells in the G1–G0 phase and more (*P* < 0.05) cells in the S phase relative to the control (Figures 4A,B). Stimulation with LPS showed a higher (*P* < 0.05) proportion of cells in the G0-G1 phase and a lower (*P* < 0.05) proportion of cells in the S phase, but the LSB group reversed (*P* < 0.05) the LPS-induced cell cycle arrest. The BREC pretreatment with SB exhibited an increased (*P* < 0.05) cyclin D1, CDK4, and CDK6 mRNA expression compared with the control; LPS treatment showed a significant decrease (*P* < 0.05) in the expression of these genes compared to the control group, while the LSB group increased (*P* < 0.05) the expression of the above genes compared with the LPS group (Figure 4C).

Moreover, stimulation with LPS a more (*P* < 0.05) significant apoptosis rate compared with control group (Figures 4D,E). Pretreatment SB decreased (*P* < 0.05) apoptosis rate compared with LPS group. In addition, Caspase 3, Caspase 9, and Bax mRNA expression was enhanced (*P* < 0.05) and Bcl-2 was decreased (*P* < 0.05) in the LPS group relative to the control group. Interestingly, the LSB group decreased (*P* < 0.05) the expression of genes related to apoptosis compared with the LPS group (Figure 4F).

### Effect of Sodium Butyrate Supplementation on Expression of Genes Related to Volatile Fatty Acid Uptake and Metabolism of Bovine Rumen Epithelial Cells Challenged With Lipopolysaccharide

**FIGURE 5:**
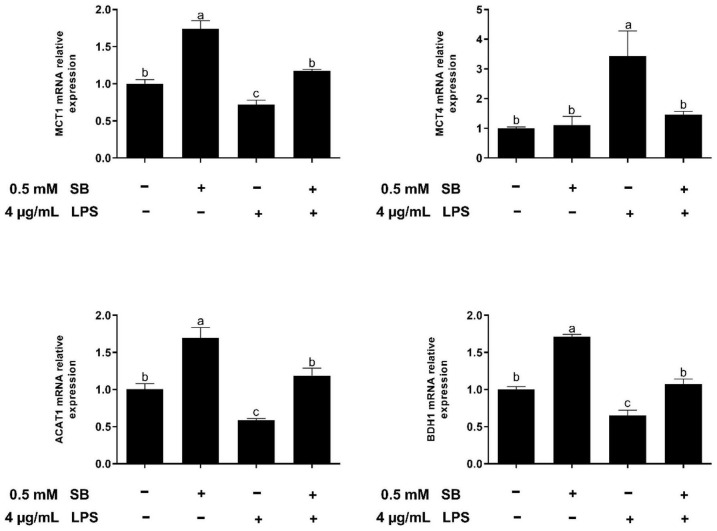
The effect of SB on the expression of genes involved in SB uptake and metabolism in BRECs stimulated with LPS. BRECs were pretreat with SB (0.5 mM) for 18 h followed by stimulation with LPS for 6 h. After incubation, cells were analyzed by qRT-PCR. The values are shown as mean ± SEM (*n* = 3). The letters in superscript indicate that the difference between groups was significant (*P* < 0.05).

The results in Figure 5 show that the supplementation with SB increased (*P* < 0.05) the expression of MCT1, ACAT1, and BDH1 relative to the control group. In addition, LPS challenge upregulated (*P* < 0.05) the expression of MCT4 and downregulated (*P* < 0.05) the expression of MCT1, ACAT1, and BDH1 compared with the control group, while these changes were reversed (*P* < 0.05) in the LSB group.

### Sodium Butyrate Alleviates Inflammatory Response by Activation of G-Protein-Coupled Receptor 41 in Lipopolysaccharide-Induced Bovine Rumen Epithelial Cells

**FIGURE 6:**
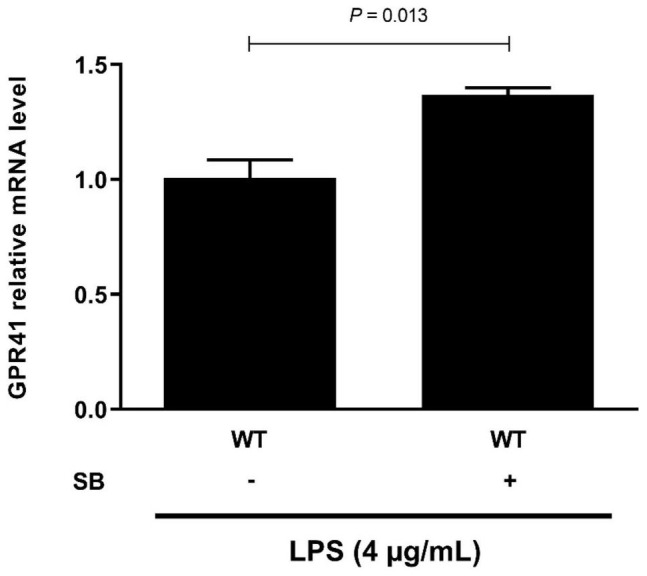
Sodium butyrate promoting the gene expression of GPR41. Generation of GPR41-knockdown (GPR41KD) BREC cell lines using the CRISPR/Cas9 system. BRECs were pretreated with SB (0 and 0.5 mM) for 18 h and then treated with 4 μg/mL LPS for 6 h. The gene expression of GPR41 was detected by qRT-PCR. The values are shown as mean ± SEM (*n* = 3).

**FIGURE 7:**
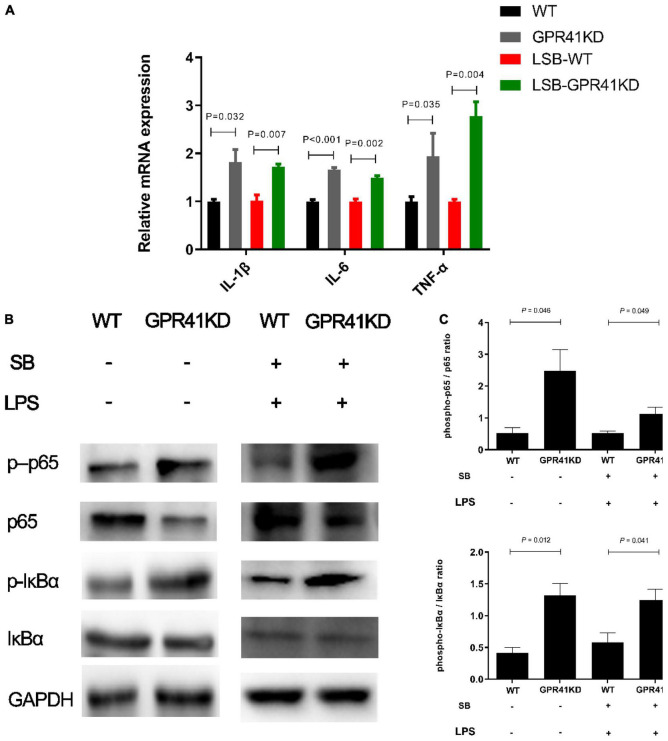
Effect of GPR41 on genes and proteins related to inflammation. Generation of GPR41-knockdown (GPR41KD) BREC cell lines using the CRISPR/Cas9 system. Pretreatment with SB in BRECs and BRECEs-GPR41KD for 18 h then treated with 4 μg/mL LPS for 6 h. After incubation, cells were analyzed by qRT-PCR and Western blotting. **(A)** Expression of genes related to the inflammatory response, normalized by GAPDH. **(B,C)** Immunoblotting and acquisition of intensity from the respective blots. The protein expression was normalized by the respective abundance of GAPDH. The values are shown as mean ± SEM (*n* = 3).

G-protein-coupled receptor 41 is the target of SCFAs, which have been shown to modulate the inflammation and immune responses (27, 28). Our results showed that SB remarkably increased (*P* < 0.05) the mRNA abundance of GPR41 (Figure 6). Moreover, GPR41KD BRECs increased (*P* < 0.05) the expression of genes and proteins related to inflammatory response compared to WT BRECs. Therefore, we hypothesized that SB may alleviate the inflammatory response by the activation of GPR41 in LPS-induced BRECs. Pretreatment with SB had no effect (*P* > 0.05) on GPR41KD BRECs (Supplementary Figures 2–4). Our results also indicated that GPR41KD BRECs enhanced (*P* < 0.05) the expression of IL-1β, IL-6, and TNF-α compared with WT BRECs, which were both treated with SB and LPS (Figure 7A). The same is true of the ratio of phosphorylated NF-κB (p65 subunit) to NF-κB (p65 subunit) and the ratio of phosphorylated IκBα to IκBα (Figures 7B,C). These results indicated that SB regulated the inflammatory response by GPR41 in LPS-induced BRECs.

### Sodium Butyrate Modulates the Expression of Genes Related to Tight Junction Proteins, and Volatile Fatty Acid Uptake and Metabolism by Activation of G-Protein-Coupled Receptor 41

**FIGURE 8:**
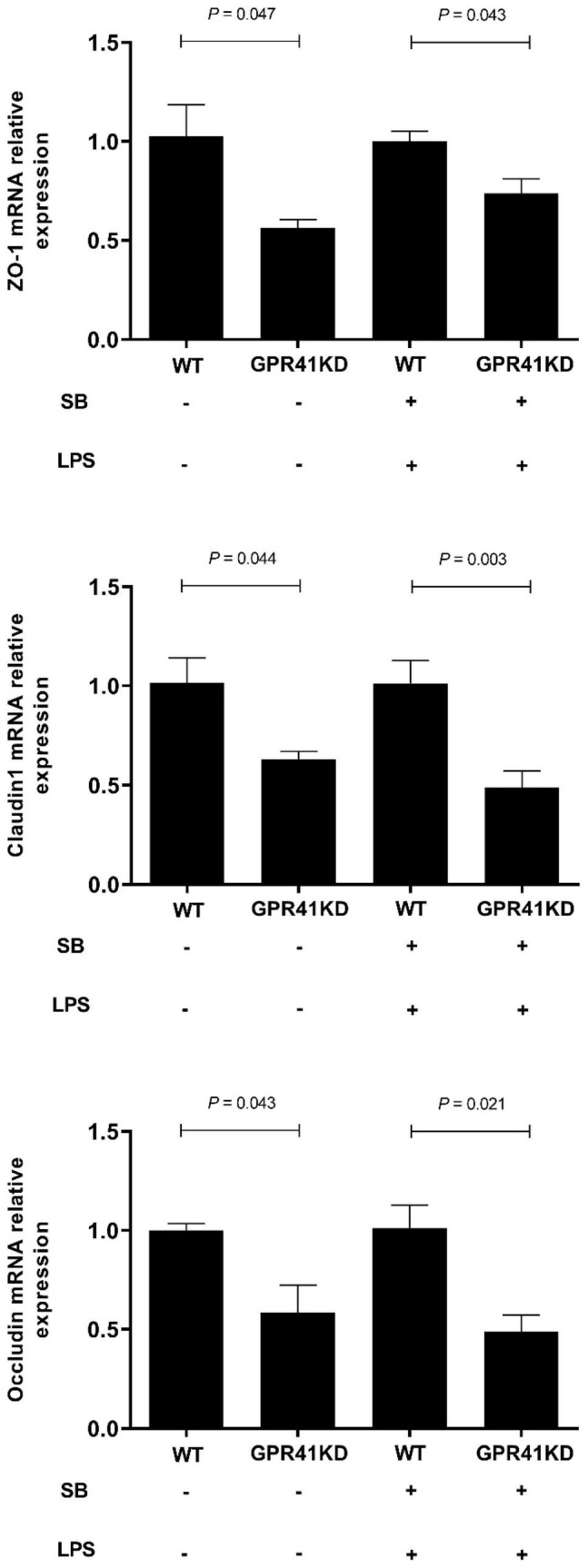
Effect of GPR41 on the gene expression of tight junction proteins. Generation of GPR41-knockdown (GPR41KD) BREC cell lines using the CRISPR/Cas9 system. Pretreatment with sodium butyrate (SB) in BRECs and BRECEs–GPR41KD for 18 h, then treated with 4 μg/mL LPS for 6 h. After incubation, cells were analyzed by qRT-PCR. The values are shown as mean ± SEM (*n* = 3).

**FIGURE 9:**
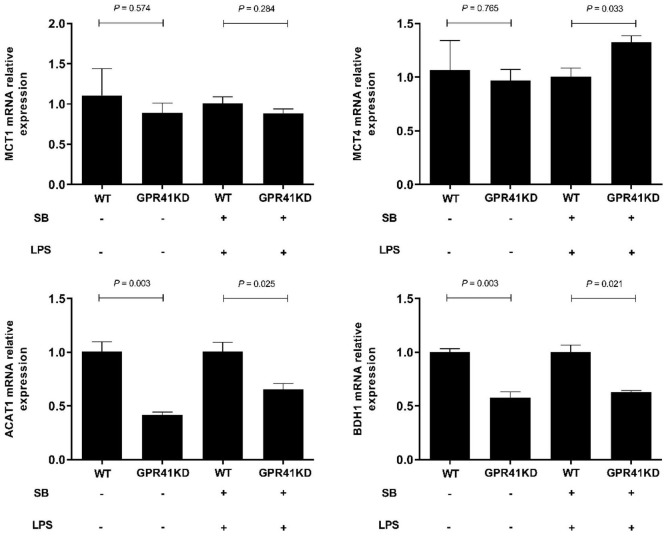
The effect of GPR41 on the expression of genes involved in SB uptake and metabolism. Generation of GPR41-knockdown (GPR41KD) BREC cell lines using the CRISPR/Cas9 system. Pretreatment with SB in BRECs and BRECEs-GPR41KD for 18 h, then treated with 4 μg/mL LPS for 6 h. After incubation, cells were analyzed by qRT-PCR. The values are shown as mean ± SEM (*n* = 3).

The expression of genes related to tight junction proteins is shown in Figure 8. Compared to WT BRECs, GPR41KD BRECs decreased (*P* < 0.05) the expression of gene related to tight junction Proteins, such as ZO-1, Claudin1, and Occludin. Similarly, compared to that of LSB-WT group, mRNA expression of these three enzymes were remarkably reduced by LSB-GPR41 group. The results in Figure 9 show that the GPR41KD BRECs induced a decrease (*P* < 0.05) in ACAT1, and BDH1 gene expression compared with WT BRECs. LSB-GPR41KD group decreased the expression of ACAT1 and BDH1, while they increased (*P* < 0.05) MCT4 mRNA expression compared with LSB-WT group.

### Sodium Butyrate Modulates Apoptosis and Cell Cycle by Activating G-Protein-Coupled Receptor 41 in Lipopolysaccharide-Induced Bovine Rumen Epithelial Cells

**FIGURE 10:**
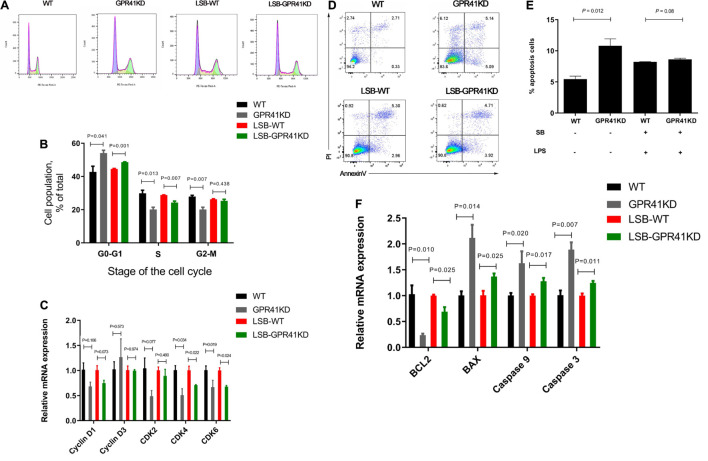
Effect of GPR41 on proliferation and apoptosis. Generation of GPR41-knockdown (GPR41KD) BREC cell lines using the CRISPR/Cas9 system. LSB-WT, pretreatment with SB in LPS-induced BRECs; LSB-GPR41KD, pretreatment with SB in LPS-induced BRECEs-GPR41KD. After incubation, cells were analyzed by flow cytometry and qRT-PCR. **(A)** A representative histogram of flow cytometric analyses. **(B)** Percentages of cells at different phases of the cell cycle. **(C)** Gene expression of Cyclin D1, Cyclin D3, CDK2, CDK4, and CDK6. **(D)** Absorbance of FITC-A; absorbance of fluorescein isothiocyanate-Annexin V. **(E)** Apoptosis percentages. **(F)** Gene expression of Caspase 3, Caspase 9, Bcl-2, and BAX. The values are shown as mean ± SEM (*n* = 3).

We then verified that SB modulates the apoptosis and cell cycle of LPS-induced BRECs by activating GPR41. In comparison with the WT BRECs, GPR41KD BRECs included a higher (*P* < 0.05) proportion of cells in the G1–G0 phase and a lower (*P* < 0.05) proportion of cells in the S and G2-M phase. Meanwhile, the LSB-GPR41 group had more (*P* < 0.05) cells in the G1-G0 phase and fewer (*P* < 0.05) cells in the S phase relative to BRECs with SB and LPS treatment (Figures 10A,B). These data suggest that lowering GPR41 expression can disrupt of progression from G1 to S phase of the cell cycle. To understand the molecular mechanisms, expression of cell cycle regulators were analyzed by qRT-PCR. In our study, GPR41KD BRECs exhibited the decreased CDK4 and CDK6 mRNA expression compared with the WT BRECs. In addition, the expression of CDK4 and CDK6 was decreased (*P* < 0.05) in GPR41KD BRECs treated with SB and LPS, while the cyclin D1 mRNA expression tended to have a decrease (*P* < 0.1) compared with WT BRECs (Figure 10C).

We found that, compared with WT BRECs, the apoptosis rate was significantly enhanced (*P* < 0.05) in GPR41KD BRECs. The LSB-GPR41KD group tended to have an increase in (*P* < 0.1) apoptosis rate compared with the LSB group (Figures 10D,E). In addition, the Caspase 3, Caspase 9, and Bax mRNA expression were enhanced (*P* < 0.05), while Bcl-2 expression was decreased (*P* < 0.05) in the WT BRECs relative to the GPR41KD BRECs (Figure 10F). The expression of genes involved in apoptosis remained at levels similar to those observed in cells treatment with LPS and SB.

## Discussion

In the present study, we demonstrated the protective effect of SB on LPS-induced BREC inflammation and the ruminal epithelium barrier, with a reduction in proinflammatory cytokine expression and a promotion of tight junction protein expression. Pretreatment with SB decreased the apoptosis rate and modulated the cell cycle. In addition, LPS-induced alteration in the expression of mRNA-related VFA uptake and metabolism was also reversed. GPR41, as the main receptor of SB, plays an important role in regulation of immune responses and epithelial barrier function (13). Our data showed that SB protected against LPS-induced BERC damage by the activation of GPR41.

The SB has been shown to strengthen the integrity of the epithelial barrier by upregulating and reorganizing the tight junction proteins that connect epithelial cells (29–31). Tight junctions play a vital role in forming a selective barrier via direct cell–cell interactions. It has been shown that LPS increases tight junction permeability, and interruption of the tight junction barrier causes it to become fragile (32). As is known, ZO-1, Occludin, and Claudin-1 are vital for tight junctions to maintain epithelial permeability (33). In the present study, we determined that SB can improve ruminal epithelial barrier function by enhancing tight-junction mRNA expression. Once rumen epithelial barrier function is damaged, rumen epithelial permeability is increased and LPS has the opportunity to translocate into the bloodstream, giving rise to a systemic inflammation response (34). The rumen is the first organ affected by LPS, which causes an inflammatory response. Cytokines act as intercellular chemical messengers involved in the regulation of immune homeostasis and inflammatory responses against different infections (35). Among the cytokines, IL-1β, IL-6, and TNF-α are considered pro-inflammatory, with one study reporting the upregulation of genes related to pro-inflammatory response during high-concentrate-diet-induced inflammation (8). Zhao et al. demonstrated that the abundance of these cytokines in BRECs induced by LPS also increases *in vitro* (9). In the present study, LPS induced an increase in proinflammatory cytokine expression. This further mediated the damage caused by inflammation in the ruminal epithelium. SB has been demonstrated to have an anti-inflammatory effect in colon diseases, and was also reported to attenuate the high-concentrate-diet-induced inflammation in rumen epithelium tissue (8). The downregulation of IL-1β, IL-6, and TNF-α in LPS-induced BRECs that were pretreated with SB emphasized the positive effect of SB on the inflammatory responses of BRECs. Our data are consistent with previous studies in bovine hepatocytes and bovine mammary epithelial cells (14, 15), which indicated that SB could protect against LPS-induced inflammation in ruminal epithelium tissue.

The NF-κB protein is a nuclear transcription factor that plays a crucial role in the innate immune system. Activation of NF-κB is involved in inflammatory processes associated with the development of mastitis (36). Upon stimulation of cells by a variety of chemical and mechanical signals that lead to the phosphorylation and degradation of IκBα. The NF-κB is phosphorylated and translocated into the nucleus, triggering the transcription of inflammatory cytokines (37). Thus, inhibition of NF-κB activation has attracted attention as a therapeutic approach intervention during immune and inflammatory events (38). The anti-inflammatory response potential of SB stems from its ability to prevent the phosphorylation and degradation of IκBα and attenuate the DNA-binding activity of NF-κB in bovine mammary epithelial cells and hepatocytes (14, 15). Our present study found that the role of SB is to suppress the translocation of phosphorylated NF-κB p65 into the nucleus. Moreover, the inhibition of NF-κB activity is most likely regulated through the decreased phosphorylation of IκBα, as shown by immunoblotting results. These results indicated that SB pretreatment ameliorates the LPS-induced inflammatory response, which is also associated with the inhibition of NF-κB activation.

In fact, apoptosis is a physiological process of cell self-destruction, including condensation of nuclear chromatin, formation of cell membrane vesicles, and cell contraction (39). NF-κB can regulate cellular apoptosis and differentiation. The previous study showed that under certain circumstances NF-κB activation is required for cell apoptosis (40). This effect involves that a pro-apoptotic and an anti-apoptotic NF-κB transcriptional target gene were Bax and Bcl-2, respectively (41). Our results indicated that the LSB group upregulated Bcl-2 (anti-apoptotic factor) and downregulated Bax (pro-apoptotic factor). Anti-apoptotic Bcl-2 can inhibit apoptosis by a caspase 9–caspase 3 pathway (42, 43). Caspase molecules can modulate death-receptor-induced and mitochondrial-induced apoptotic pathways (40, 44). Caspase 3 and Caspase 9 mRNA expression decreased with SB pretreatment in LPS-induced BRECs. Furthermore, pretreatment with SB exhibited less apoptosis in LPS-induced BRECs. The attenuating effect of SB on LPS-induced apoptosis was further confirmed, and apoptosis and the cell cycle were closely related. Inhibition of caspase 3 prevents cell cycle arrest has been investigated (45). The current results showed that SB promoted cell-cycle progression by increasing the cellular fraction of the G0/G1 phase to S phase transition, which was consistent with previous studies (46). We also demonstrated that cyclin D1, CDK4, and CDK6 mRNA expression was increased in the LSB group compared with the LPS group. Malhi et al. reported that the gene expression of cyclin D1 and CDK4 was increased by treatment with exogenous butyrate in ruminal epithelial cells of goats (46). In addition, our results are consistent with previous study that infusions of SB can inhibit apoptosis and promote proliferation in rumen epithelium tissue (17). Therefore, SB protects against LPS-induced BERC damage by protecting barrier function, alleviating the inflammatory response, and regulating cell cycle and apoptosis. To the best of our knowledge, although SB is known to protect against LPS-induced BERC damage, there is a dearth of information on the molecular. However, butyrate acting as a ligand to activate GPR41 and modulate inflammatory response has been well characterized. Trompette et al. described increased inflammation in GPR41 knockout mice (47). In our study, we found that SB improved the gene expression of GPR41. In our previous study, rumen epithelial barrier and proinflammatory response injury can occur if GPR41 is deficient in BRECs (13), which is consistent with the present study. Liu et al. reported that SCFA acetate upregulated GPR41 and inhibited the NF-κB pathway in amyloid-β-induced BV2 microglia (48). In the current study, we found that SB inhibited NF-κB activation in LPS-induced BRECs, and this activation was reversed in GPR41KD-BRECs, which indicated that SB inhibited NF-κB activation in LPS-induced BRECs through the GPR41 receptor. We then verified that SB-promoted cell-cycle progression and reduced apoptosis rate were abolished in GPR41KD BRECs. In addition, SB-attenuated neuronal apoptosis via the GPR41 pathway has been demonstrated (49). Taken together, SB protects against LPS-induced BERCs damage via the GPR41 pathway.

A state of inflammation is not only largely associated with metabolism disruption, it may also alter nutrient transport by epithelial cells (18, 50). The main metabolism of rumen epithelial tissue is the ketogenic effect, which converts VFA into ketone bodies to provide energy for the body (51). Therefore, we determined the expression of genes related to VFA uptake and metabolism in LPS. MCT1, localized on the basolateral side of rumen epithelium tissue, is a main transporter for SB (52, 53). In addition, butyrate-induced increases in the expression and resulting activity of MCT1 serve as a mechanism to maximize the intracellular availability of butyrate. Treatment with SB led to a concentration- and time-dependent upregulation of MCT1 mRNA in colonic epithelial cells (53). However, inflammation can lead to the dysregulation of MCT1 (54). In the present study, LPS decreased the expression of MCT1, while the mRNA expression of MCT1 increased in the LSB group, which indicated that inflammation caused the dysregulation of MCT1, but SB alleviates this effect. In addition, MCT4 expression was increased upon exposure to LPS. This is consistent with the observed results of Dennis et al. (18). The higher expression of MCT4 is associated with a greater content of lactate (55). Inflammation can increase glycolysis, leading to mediating lactate accumulation and then upregulating of MCT4. Pretreatment with SB relieved irritation, and MCT4 was downregulated. In addition, this effect was abolished in GPR41KD BRECs, which may indicate that MCT4 expression is strongly linked to inflammation. As further evidence for LPS influencing ketogenic metabolism in the present study, LPS induced a decrease in the expression of ACAT1 and BDH1, which indicated a shift away from ketogenesis. LPS-stimulated inflammation can cause a decline in the production of ketone bodies in mice (56). In addition, increases in butyrate concentration in calf rumens stimulates changes in the expressions of genes and proteins involved in the ketogenesis pathway (57). In this study, we proved that pretreatment with SB restored the LPS-induced expression of ACAT1, and BDH1 expression decreased. Knockdown experiments demonstrated that SB promoted ketone body formation by activating the GPR41 pathway.

In conclusion, our data showed that SB possesses ruminal epithelial barrier- maintaining capability and anti-inflammation in BRECs. Activated GPR41 may play a vital role in the regulation of the expression of inflammatory and ketogenic-related genes. Taken together, we conclude that SB may be a promising additive as a treatment to ameliorate the LPS-related induction of ruminal inflammation in dairy cows.

## Data Availability Statement

The original contributions presented in the study are included in the article/Supplementary Material, further inquiries can be directed to the corresponding authors.

## Ethics Statement

The animal study was reviewed and approved by Yangzhou University.

## Author Contributions

TY performed the experimental work, analyzed the data, and wrote the manuscript. OD performed the experimental work. MJ and XM revised the manuscript. KZ contributed to the experimental idea, wrote, reviewed, and edited the manuscript. GZ provided the support of funding. All authors contributed to the article and approved the submitted version.

## Conflict of Interest

The authors declare that the research was conducted in the absence of any commercial or financial relationships that could be construed as a potential conflict of interest.

## Publisher’s Note

All claims expressed in this article are solely those of the authors and do not necessarily represent those of their affiliated organizations, or those of the publisher, the editors and the reviewers. Any product that may be evaluated in this article, or claim that may be made by its manufacturer, is not guaranteed or endorsed by the publisher.

## Footnotes

1
http://blooge.cn/RefFinder/

## References

[1] Pan XH Yang L Xue FG Xin HR Jiang LS Xiong BH Relationship between thiamine and subacute ruminal acidosis induced by a high-grain diet in dairy cows. *J Dairy Sci.* (2016) 99:8790–801. 10.3168/jds.2016-10865 27568043

[2] Steele MA Croom J Kahler M AlZahal O Hook SE Plaizier K Bovine rumen epithelium undergoes rapid structural adaptations during grain-induced subacute ruminal acidosis. *Am J Physiol Regul Integr Comp Physiol.* (2011) 300:1515–23. 10.1152/ajpregu.00120.2010 21451145

[3] Penne GB Oba M Gabel G Aschenbach JR. A single mild episode of subacute ruminal acidosis does not affect ruminal barrier function in the short term. *J Dairy Sci.* (2010) 93:4838–45. 10.3168/jds.2010-3406 20855017

[4] Khafipour E Krause DO Plaizier JC. Alfalfa pellet-induced subacute ruminal acidosis in dairy cows increases bacterial endotoxin in the rumen without causing inflammation. *J Dairy Sci.* (2009) 92:1712–24. 10.3168/jds.2008-1656 19307653

[5] Gareau MG Silva MA Perdue MH. Pathophysiological mechanisms of stress-induced intestinal damage. *Curr Mol Med.* (2008) 8:274–81. 10.2174/156652408784533760 18537635

[6] Gozho GN Krause DO Plaizier C. Ruminal lipopolysaccharide concentration and inflammatory response during grain-induced subacute ruminal acidosis in dairy cows. *J Dairy Sci.* (2007) 90:856–66. 10.3168/jds.S0022-0302(07)71569-2 17235162

[7] Emmanuel DG Dunn SM Ametaj BN. Feeding high proportions of barley grain stimulates an inflammatory response in dairy cows. *J Dairy Sci.* (2008) 91:606–14. 10.3168/jds.2007-0256 18218747

[8] Dai H Liu X Yan J Aabdin ZU Bilal MS Shen X. Sodium butyrate ameliorates high-concentrate diet-induced inflammation in the rumen epithelium of dairy goats. *J Agric Food Chem.* (2017) 65:596–604. 10.1021/acs.jafc.6b04447 28032994

[9] Zhao CX Wang YZ Yuan X Sun GQ Shen BY Xu F Berberine inhibits lipopolysaccharide-induced expression of inflammatory cytokines by suppressing TLR4-mediated NF-κB and MAPK signaling pathways in rumen epithelial cells of Holstein calves. *J Dairy Res.* (2019) 86:171–6. 10.1017/S0022029919000323 31142385

[10] Chen C Yu Z Li Y Fichna J Storr M. Effects of berberine in the gastrointestinal tract - a review of actions and therapeutic implications. *Am J Chin Med.* (2014) 42:1053–70. 10.1142/S0192415X14500669 25183302

[11] Uchiyama R Chassaing B Zhang B Gewirtz AT. MyD88-mediated TLR signaling protects against acute rotavirus infection while inflammasome cytokines direct Ab response. *Innate Immun.* (2015) 21:416–28. 10.1177/1753425914547435 25213347 PMC7534410

[12] Vadiveloo PK. Macrophages–proliferation, activation, and cell cycle proteins. *J Leukoc Biol.* (1999) 66:579–82. 10.1002/jlb.66.4.579 10534112

[13] Zhan K Gong X Chen Y Jiang M Yang T Zhao G. Short-Chain Fatty Acids Regulate the Immune Responses via G Protein-Coupled Receptor 41 in Bovine Rumen Epithelial Cells. *Front Immunol.* (2019) 10:2042. 10.3389/fimmu.2019.02042 31555273 PMC6722193

[14] Sun X Luo S Jiang C Tang Y Cao Z Jia H Sodium butyrate reduces bovine mammary epithelial cell inflammatory responses induced by exogenous lipopolysaccharide, by inactivating NF-kappaB signaling. *J Dairy Sci.* (2020) 103:8388–97. 10.3168/jds.2020-18189 32622605

[15] Xu T Ma N Wang Y Shi X Loo JJ Sodium butyrate supplementation alleviates the adaptive response to inflammation and modulates fatty acid metabolism in lipopolysaccharide-stimulated bovine hepatocytes. *J Agric Food Chem.* (2018) 66:6281–90. 10.1021/acs.jafc.8b01439 29877079

[16] Machado RA Constantino SL Tomasi CD Rojas HA Vuolo FS Vitto MF Sodium butyrate decreases the activation of NF-kappaB reducing inflammation and oxidative damage in the kidney of rats subjected to contrast-induced nephropathy. *Nephrol Dial Transplant.* (2012) 27:3136–40. 10.1093/ndt/gfr807 22273669

[17] Liu L Sun D Mao S Zhu W Liu J. Infusion of sodium butyrate promotes rumen papillae growth and enhances expression of genes related to rumen epithelial VFA uptake and metabolism in neonatal twin lambs. *J Anim Sci.* (2019) 97:909–21. 10.1093/jas/sky459 30535158 PMC6377441

[18] Kent-Dennis C Penner GB. Effects of a proinflammatory response on metabolic function of cultured, primary ruminal epithelial cells. *J Dairy Sci.* (2021) 104:1002–17. 10.3168/jds.2020-19092 33131809

[19] Brown AJ Goldsworthy SM Barnes AA Eilert MM Tcheang L Daniels D The Orphan G protein-coupled receptors GPR41 and GPR43 are activated by propionate and other short chain carboxylic acids. *J Biol Chem.* (2003) 278:11312–9. 10.1074/jbc.M211609200 12496283

[20] Poul EL Loison C Struyf S Springael JY Lannoy V Decobecq ME Functional characterization of human receptors for short chain fatty acids and their role in polymorphonuclear cell activation. *J Biol Chem.* (2003) 278:25481–9. 10.1074/jbc.M301403200 12711604

[21] Nilsson NE Kotarsky K Owman C Olde B. Identification of a free fatty acid receptor, FFA2R, expressed on leukocytes and activated by short-chain fatty acids. *Biochem Biophys Res Commun.* (2003) 303:1047–52. 10.1016/s0006-291x(03)00488-1 12684041

[22] Sina C Gavrilova O Forster M Till A Derer S Hildebrand F G protein-coupled receptor 43 is essential for neutrophil recruitment during intestinal inflammation. *J Immunol.* (2009) 183:7514–22. 10.4049/jimmunol.0900063 19917676

[23] Maslowski KM Vieira AT Ng A Kranich J Sierro F Yu D Regulation of inflammatory responses by gut microbiota and chemoattractant receptor GPR43. *Nature.* (2009) 461:1282–6. 10.1038/nature08530 19865172 PMC3256734

[24] Xiong Y Miyamoto N Shibata K Valasek MA Motoike T Kedzierski RM Short-chain fatty acids stimulate leptin production in adipocytes through the G protein-coupled receptor GPR41. *Proc Natl Acad Sci USA.* (2004) 101:1045–50. 10.1073/pnas.2637002100 14722361 PMC327148

[25] Bindels LB Em Dewulf NM. Delzenne. GPR43/FFA2: physiopathological relevance and therapeutic prospects. *Trends Pharmacol Sci.* (2013) 34:226–32. 10.1016/j.tips.2013.02.002 23489932

[26] Livak KJ Schmittgen TD. Analysis of relative gene expression data using real-time quantitative PCR and the 2(-Delta Delta C(T)) Method. *Methods.* (2001) 25:402–8. 10.1006/meth.2001.1262 11846609

[27] Ang Z Ding JL. GPR41 and GPR43 in obesity and inflammation - protective or causative? *Front Immunol.* (2016) 7:28. 10.3389/fimmu.2016.00028 26870043 PMC4734206

[28] Correa-Oliveira R Fachi JL Vieira A Sato FT Vinolo MA. Regulation of immune cell function by short-chain fatty acids. *Clin Transl Immunol.* (2016) 5:e73. 10.1038/cti.2016.17 27195116 PMC4855267

[29] Mariadason JM Barkla DH Gibson PR. Effect of short-chain fatty acids on paracellular permeability in Caco-2 intestinal epithelium model. *Am J Physiol.* (1997) 0272:G705–12. 10.1152/ajpgi.1997.272.4.G705 9142899

[30] Mariadason JM Catto-Smith A Gibson PR. Modulation of distal colonic epithelial barrier function by dietary fibre in normal rats. *Gut.* (1999) 44:394–9. 10.1136/gut.44.3.394 10026327 PMC1727405

[31] Ohata A Usami M Miyoshi M. Short-chain fatty acids alter tight junction permeability in intestinal monolayer cells via lipoxygenase activation. *Nutrition.* (2005) 21:838–47. 10.1016/j.nut.2004.12.004 15975492

[32] Ma TY Iwamoto GK Hoa NT Akotia V Pedram A Boivin MA TNF-alpha-induced increase in intestinal epithelial tight junction permeability requires NF-kappa B activation. *Am J Physiol Gastrointest Liver Physiol.* (2004) 286:G367–76. 10.1152/ajpgi.00173.2003 14766535

[33] Anderson JM Itallie CMV. Tight junctions and the molecular basis for regulation of paracellular permeability. *Am J Physiol.* (1995) 269:G467–75. 10.1152/ajpgi.1995.269.4.G467 7485497

[34] Baumann H Gauldie J. The acute phase response. *Immunol Today.* (1994) 15:74–80. 7512342 10.1016/0167-5699(94)90137-6

[35] Okada H Ohtsuka H Nai S Kon Kirisawa R Yokomizo Y Yoshino T Effects of lipopolysaccharide on production of interleukin-1 and interleukin-6 by bovine mammary epithelial cells in vitro. *J Vet Med Sci.* (1999) 61:33–5. 10.1292/jvms.61.33 10027160

[36] Gong XX Su XS Zhan K Zhao GQ. The protective effect of chlorogenic acid on bovine mammary epithelial cells and neutrophil function. *J Dairy Sci.* (2018) 101:10089–97. 10.3168/jds.2017-14328 30146292

[37] Niederberger E Geisslinger G. The IKK-NF-kappaB pathway: a source for novel molecular drug targets in pain therapy? *Faseb J.* (2008) 22:3432–42. 10.1096/fj.08-109355 18559989

[38] Calzado MA Bacher S Schmitz ML. NF-kappaB inhibitors for the treatment of inflammatory diseases and cancer. *Curr Med Chem.* (2007) 14:367–76. 10.2174/092986707779941113 17305539

[39] Holmgren L Reilly MSO Folkman J. Dormancy of micrometastases: balanced proliferation and apoptosis in the presence of angiogenesis suppression. *Nat Med.* (1995) 1:149–53. 10.1038/nm0295-149 7585012

[40] Kucharczak J Simmons MJ Fan Y Gélinas C. To be, or not to be: NF-kappaB is the answer—role of Rel/NF-kappaB in the regulation of apoptosis. *Oncogene.* (2003) 22:8961–82. 10.1038/sj.onc.1207230 14663476

[41] Li L Wu WJ Huang WJ Hu G Yuan WF Li WF. NF-K b RNAi decreases the Bax/Bcl-2 ratio and inhibits TNF-α-induced apoptosis in human alveolar epithelial cells. *Inflamm Res.* (2013) 62:387–97. 10.1007/s00011-013-0590-7 23334076

[42] McNutt MC Lagace TA Horton JD. Catalytic activity isnot required for secreted PCSK9 to reduce low density lipoprotein receptors in HepG2 cells. *J Biol Chem.* (2007) 282:20799–803. 10.1074/jbc.C700095200 17537735 PMC13276728

[43] Wu CY Tang ZH Jiang L Li XF Jiang ZS Liu LS. PCSK9 siRNA inhibits HUVEC apoptosis induced by ox-LDL via Bcl/Bax-caspase9-caspase3 pathway. *Mol Cell Biochem.* (2012) 359:347–58. 10.1007/s11010-011-1028-6 21847580

[44] Nagata S. Apoptosis by death factor. *Cell.* (1997) 88:355–65. 10.1016/s0092-8674(00)81874-7 9039262

[45] Song FM Yu XY Zhong T Wang ZY Meng XL Li ZL Caspase-3 Inhibition Attenuates the Cytopathic Effects of EV71 Infection. *Front Microbiol.* (2018) 26:817. 10.3389/fmicb.2018.00817 29755438 PMC5932146

[46] Malhi M Gui H Yao L Aschenbach JR Gabel G Shen Z. Increased papillae growth and enhanced short-chain fatty acid absorption in the rumen of goats are associated with transient increases in cyclin D1 expression after ruminal butyrate infusion. *J Dairy Sci.* (2013) 96:7603–16. 10.3168/jds.2013-6700 24119813

[47] Trompette A Gollwitzer ES Yadava K Sichelstiel AK Sprenger N Ngom-Bru C Gut microbiota metabolism of dietary fiber influences allergic airway disease and hematopoiesis. *Nat Med.* (2014) 20:159–66. 10.1038/nm.3444 24390308

[48] Liu J Li H Gong T Chen W Mao S Kong Y Anti-neuroinflammatory Effect of Short-Chain Fatty Acid Acetate against Alzheimer’s Disease via Upregulating GPR41 and Inhibiting ERK/JNK/NF-kappaB. *J Agric Food Chem.* (2020) 68:7152–61. 10.1021/acs.jafc.0c02807 32583667

[49] Zhou Z Xu N Matei N McBride DW Ding Y Liang H Sodium butyrate attenuated neuronal apoptosis via GPR41/Gbetagamma/PI3K/Akt pathway after MCAO in rats. *J Cereb Blood Flow Metab.* (2021) 41:267–81. 10.1177/0271678X20910533 32151222 PMC8370004

[50] Peuhkuri K Vapaatalo H Korpela R. Even low-grade inflammation impacts on small intestinal function. *World J Gastroenterol.* (2010) 6:1057–62. 10.3748/wjg.v16.i9.1057 20205274 PMC2835780

[51] Allen MS. Drives and limits to feed intake in ruminants. *Anim Prod Sci.* (2014) 54:1513–24. 10.1071/an14478

[52] Muller F Huber K Pfannkuche H Aschenbach JR Breves G Gabel G. Transport of ketone bodies and lactate in the sheep ruminal epithelium by monocarboxylate transporter 1. *Am J Physiol Gastrointest Liver Physiol.* (2002) 283:G1139–46. 10.1152/ajpgi.00268.2001 12381528

[53] Cuff MA Lambert DW Shirazi-Beechey SP. Substrate-induced regulation of the human colonic monocarboxylate transporter, MCT1. *J Physiol.* (2002) 539:361–71. 10.1113/jphysiol.2001.014241 11882670 PMC2290148

[54] Thibault R Coppet PD Daly K Bourreille A Cuff M Bonnet C Down-regulation of the monocarboxylate transporter 1 is involved in butyrate deficiency during intestinal inflammation. *Gastroenterology.* (2007) 133:1916–27. 10.1053/j.gastro.2007.08.041 18054563

[55] Hu WS Dodge TC Frame KK Himes VB. Effect of glucose on the cultivation of mammalian cells. *Dev Biol Stand.* (1987) 66:279–90. 3582758

[56] Memon RA Feingold KR Moser AH Doerrler W Adi S Dinarello CA Differential effects of interleukin-1 and tumor necrosis factor on ketogenesis. *Am J Physiol.* (1992) 263:301–9. 10.1152/ajpendo.1992.263.2.E301 1514611

[57] Niwinska B Hanczakowska E Arciszewski MB Klebaniuk R. Review: exogenous butyrate: implications for the functional development of ruminal epithelium and calf performance. *Animal.* (2017) 11:1522–30. 10.1017/S1751731117000167 28193308

