# Supplementary material for: Protective Roles of Sodium Butyrate in Lipopolysaccharide-Induced Bovine Ruminal Epithelial Cells by Activating G Protein-Coupled Receptors 41
Source: Front Nutr. 2022 May 6;9:842634. doi: 10.3389/fnut.2022.842634 (PMC9121101; doi:10.3389/fnut.2022.842634)

Supplementary Figure 1. Sodium butyrate (SB) inhibiting gene expression of inflammatory cytokines. The BRECs were cultured in the presence or absence of 0.5 mM SB for 18 h. The gene expression of IL-1β, IL-6, and TNF-α was analyzed by qRT-PCR, which are normalized to GAPDH. Values are shown as mean ± SEM (n = 3).

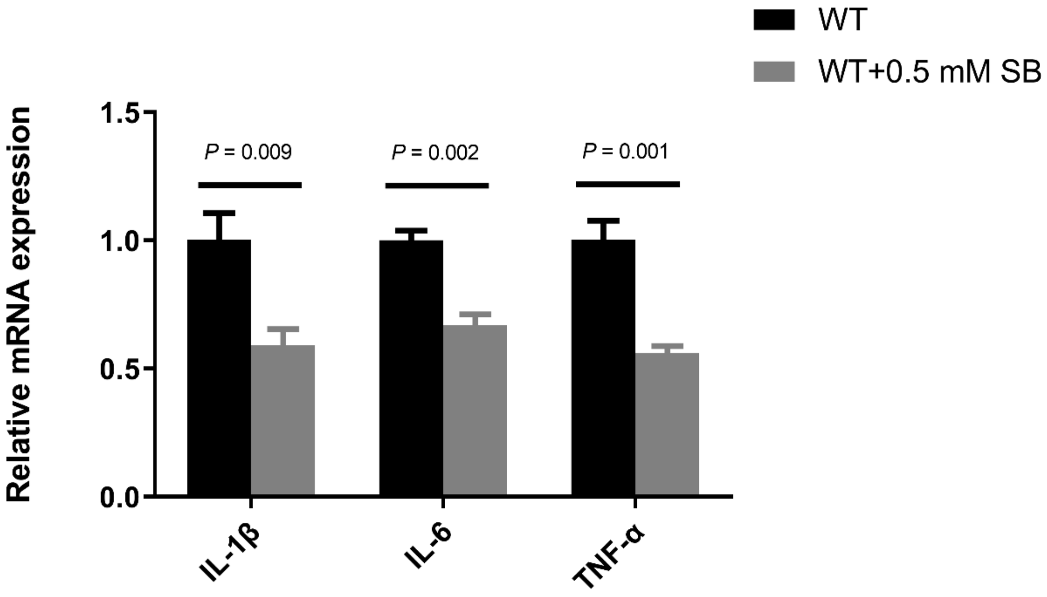

Supplementary Figure 2. Effect of Sodium butyrate (SB) on genes and proteins related to inflammation in GPR41KD BRECs. Generation of GPR41-knockdown (GPR41KD) BREC cell lines using the CRISPR/Cas9 system. The GPR41KD BRECs were cultured in the presence or absence of 0.5 mM SB for 18 h. After incubation, cells were analyzed by qRT-PCR and Western blotting. (A) Expression of genes related to the inflammatory response, normalized by GAPDH. (B, C) Immunoblotting and acquisition of intensity from the respective blots. The protein expression was normalized by the respective abundance of GAPDH. The values are shown as mean ± SEM (n = 3).

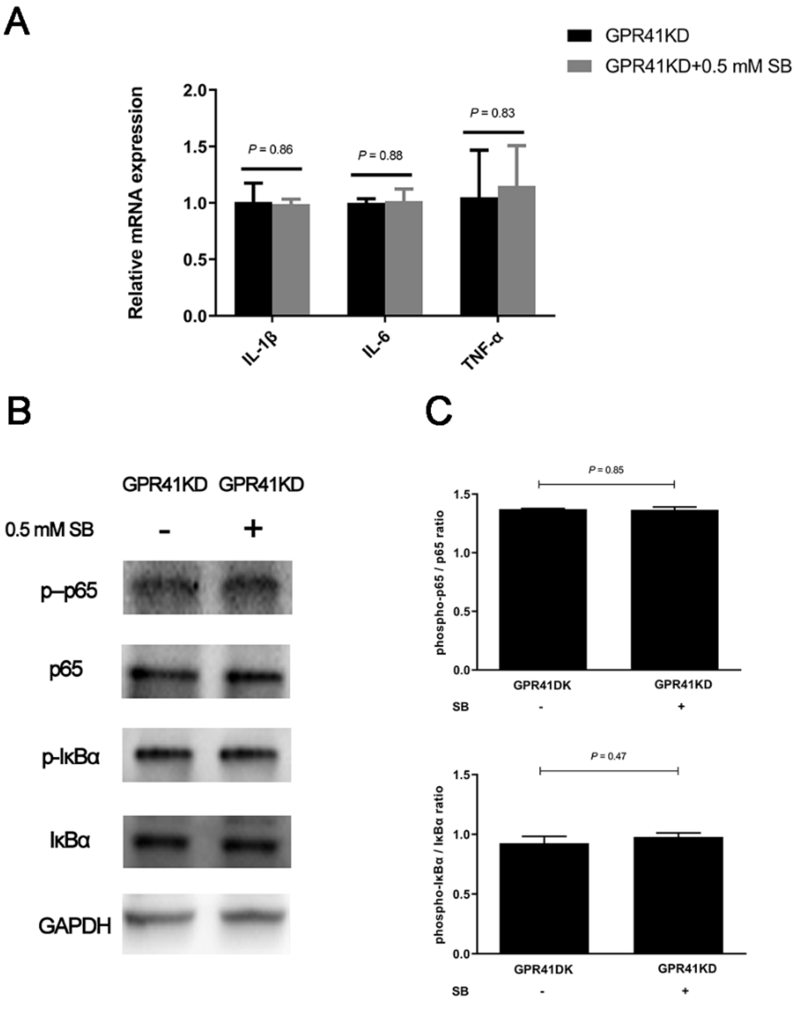

Supplementary Figure 3. The effect of sodium butyrate (SB) on the expression of genes in GPR41KD BRECs. Generation of GPR41-knockdown (GPR41KD) BREC cell lines using the CRISPR/Cas9 system. The GPR41KD BRECs were cultured in the presence or absence of 0.5 mM SB for 18 h. After incubation, cells were analyzed by qRT-PCR and Western blotting. (A) The expression of genes related to SB uptake and metabolism. (B) The expression of genes involved in tight junction proteins. (C) Gene expression of Cyclin D1, Cyclin D3, CDK 2, CDK 4, and CDK6. (D) Gene expression of Caspase 3, Caspase 9, BCL2, and BAX. The values are shown as mean ± SEM (n = 3).

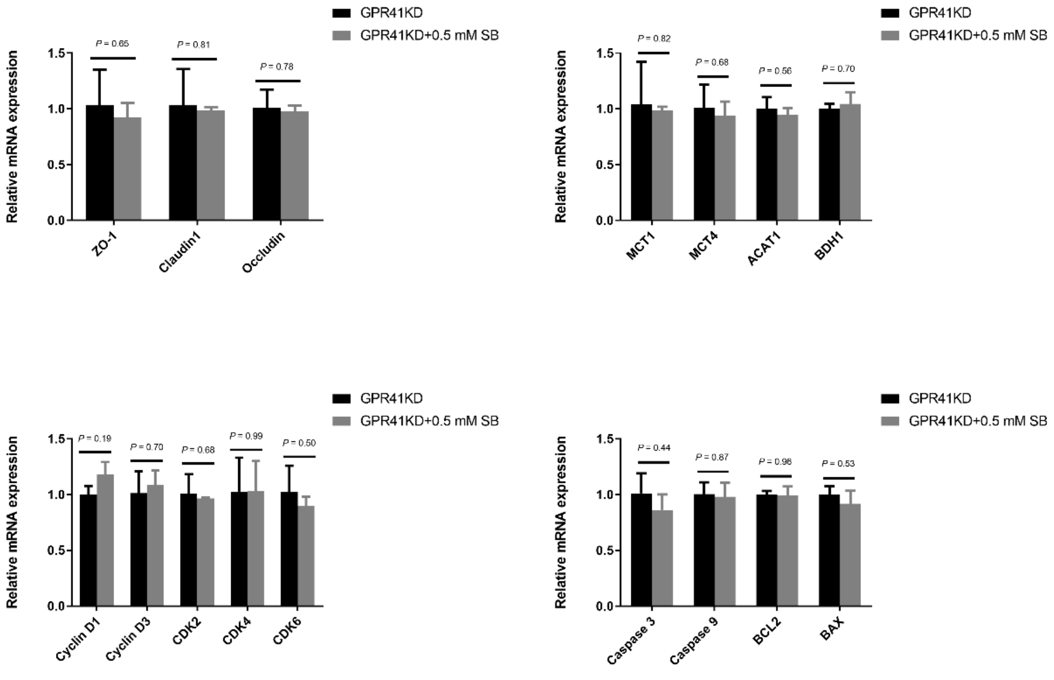

Supplementary Figure 4. Effect of sodium butyrate (SB) on proliferation and apoptosis in GPR41KD BRECs. Generation of GPR41-knockdown (GPR41KD) BREC cell lines using the CRISPR/Cas9 system. The GPR41KD BRECs were cultured in the presence or absence of 0.5 mM SB for 18 h. After incubation, cells were analyzed by flow cytometry. (A) Percentages of cells at different phases of the cell cycle. (B) Apoptosis percentages. The values are shown as mean ± SEM (n = 3).

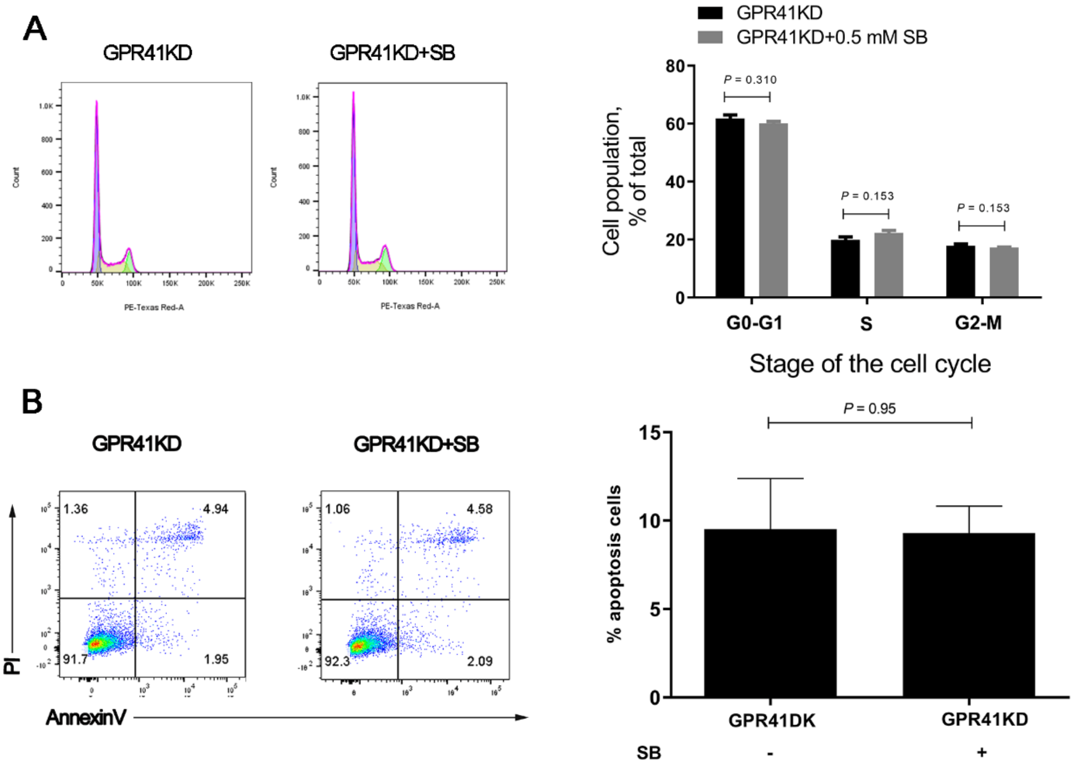

Supplementary Figure 5. The above diagram summarizes the mechanism by which protective roles of sodium butyrate in lipopolysaccharide-induced bovine ruminal epithelial cells by activating G protein-coupled receptors 41.

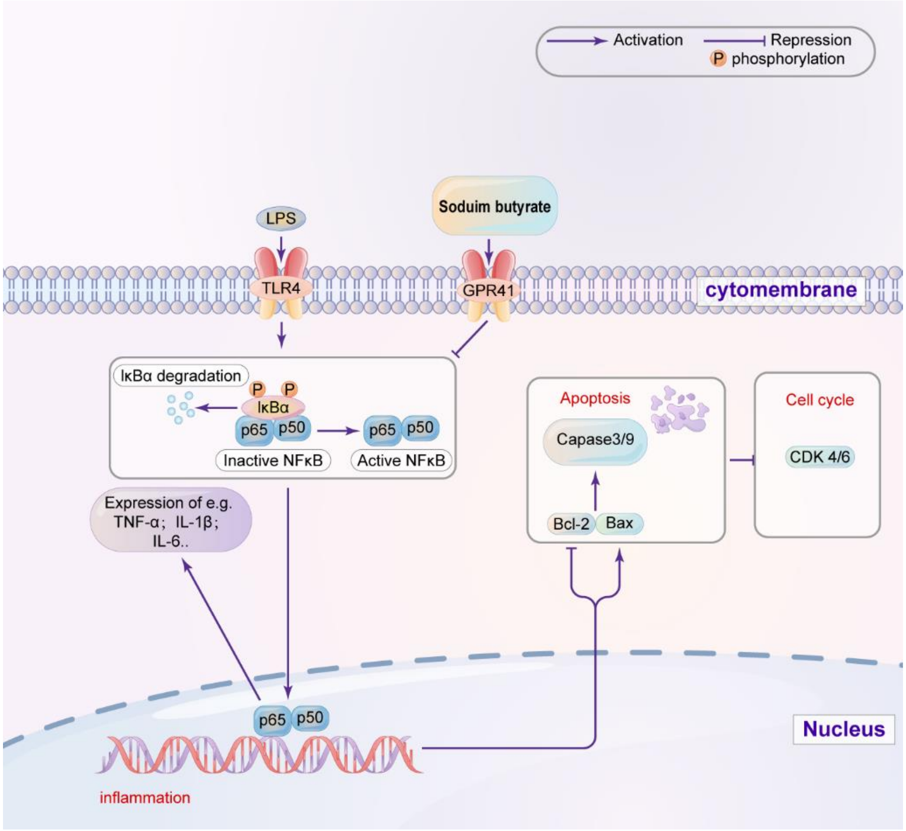

Supplement: Supplementary file 1 [file Data_Sheet_1.PDF]
